# The microbial metabolite butyrate enhances the effector and memory functions of murine CD8+ T cells and improves anti-tumor activity

**DOI:** 10.3389/fmed.2025.1577906

**Published:** 2025-06-24

**Authors:** Douglas A. Gaskarth, Shujun Fan, Andrew J. Highton, Roslyn A. Kemp

**Affiliations:** Department of Microbiology and Immunology, University of Otago, Dunedin, New Zealand

**Keywords:** butyrate, cancer, CD8+ T cells, activation, effector function, short-chain fatty acids, T cell memory, immunotherapy

## Abstract

**Introduction:**

CD8+ T cells are vital in the immune control of cancer and a key player in cell-based cancer immunotherapy. Recent studies have shown that microbial short-chain fatty acids (SCFA) can promote both effector and memory phenotypes in CD8+ T cells and may thereby enhance protection against cancer.

**Methods:**

In this study, we determined the effect of SCFA butyrate on mouse CD8+ T cell function *in vitro* and *in vivo*, using the OT-I model.

**Results:**

Butyrate co-culture with anti-CD3 + anti-CD28 activated T cells *in vitro* enhanced the frequency of effector CD8+ IFN-γ-producing cells, and the amount of cytokine produced per cell. Culture with butyrate also enhanced the activation, TCR expression, and levels of phosphorylated mTOR proteins within CD8+ T cells but reduced proliferation rate and increased apoptosis. Butyrate-treated activated cells conferred tumor protection after adoptive transfer. Butyrate-treated cells were present at higher frequencies within the tumor compared to non-butyrate treated cells, and expressed IFN-γ. When analyzed using high dimensional cytometry, the tumors of mice that received butyrate-treated cells were enriched in clusters displaying an effector memory phenotype with high expression of IL-15Rβ and T-bet.

**Discussion:**

Our findings show that butyrate promotes the effector activity of CD8+ T cells in culture, which can persist *in vivo* while also stimulating memory phenotypes. Consequently, butyrate treatment may have strong application in T cell-based immunotherapies to improve protective cell functions and patient outcomes.

## Introduction

Recently, the gut microbiota, and their metabolites, have become major areas of cancer research. Cancer continues to kill millions of people annually with limited effective treatment options and high treatment costs. The composition of the gut microbiome can directly impact the development of cancer, the efficacy of cancer therapies and the likelihood of severe side effects of treatment (1–3). Microbes have been associated with response rate to immune checkpoint blockade (ICB) therapies. A higher abundance of commensal microbes such as *Akkermansia muciniphila* and *Bifidobacterium longum* was associated with improved response rates to anti-PD-1 and anti-PD-L1 therapies in patients with epithelial tumors and metastatic melanoma (4, 5). Furthermore, response to CTLA-4 therapy required the presence of *Bacteroides thetamicron* and *Bacteroides fragilis* species in mice (6). The mechanism behind these improved response rates was modulation of the host anti-tumor immune response, particularly T cells, by microbial components. These components enhanced both the priming of T cells by antigen presenting cells and the anti-tumor function of T cells. Consequently, the use of bacterial components is now under investigation to improve immunotherapy responses.

Bacterial short chain fatty acids (SCFAs), a byproduct of the fermentation of fiber by gut microbes, may have strong applications in immunotherapy. SCFAs are abundant bacterial-derived metabolites within the mammalian gut and can modulate the activity of immune cells through G-protein coupled receptors (GPR) interactions, their function as histone deacetylase (HDAC) inhibitors, and by acting as a fuel source (7, 8). The SCFAs, pentanoate and butyrate, enhanced mTOR signaling, promoted effector cytokine production, and facilitated the generation of memory precursor T cells (9, 10). Butyrate also enhanced intracellular T cell receptor signaling, NF-κB signaling and response to anti-PD-1 therapy and chemotherapy in mice (11–13).

Despite these promising studies, a detailed description of how butyrate impacts the function of CD8+ T cells *in vitro* has not been documented, nor has the memory phenotype of butyrate-treated T cells after adoptive transfer. Luu et al. (14) identified improved anti-tumor activity of CD8+ T cells and CAR T cells following *in vitro* culture with the SCFAs, butyrate and pentanoate; however, detailed study of multiple anti-tumor functions was not provided. To address this knowledge gap, we characterized the phenotype and function of CD8+ T cells after treatment with the SCFA butyrate *in vitro*. We then explored whether *in vitro* butyrate treatment altered the memory phenotype and protective function of antigen-specific CD8+ T cells when adoptively transferred into tumor bearing mice. Butyrate treatment promoted both the generation and function of effector CD8+ T cells *in vitro*. However, these effects occurred at the expense of rapid proliferation. Butyrate-treated cells were present at higher frequencies in the tumors of mice, displayed an activated effector memory phenotype and provided enhanced anti-tumor protection compared to non-butyrate-treated cells, which extends the findings of Luu et al. (14). These data indicate that treatment with butyrate may be an effective way to improve the effector functions of CD8+ T cells and their differentiation into memory cells.

## Materials and methods

### Mice

Female 6–12 weeks old C57Bl/6J-Ptp (CD45.1) and OT-I (CD45.2) mice, originally sourced from Jackson Laboratories, were housed in SPF conditions at the Biomedical Research Facility, University of Otago. OT-I mice were used for all *in vitro* experiments. All experiments were approved by the University of Otago Animal Ethics Committee in accordance with specific animal use protocols.

### *In vitro* CD8+ T cell culture and activation

CD8+ T cells were isolated and purified from the spleens of OT-I mice using the EasySep Mouse CD8+ T cell Isolation Kit (STEMCELL Technologies, Vancouver, Canada) as per manufacturer’s instructions. 5 × 10^5^ CD8+ T cells were cultured in 24-well plates for three days at 37°C, 5% CO_2_ with RPMI, anti-CD3/anti-CD28 dynabeads (Thermofisher Scientific, Waltham, MA, USA) at a 1:1 ratio to cells, IL-2 (30 IU/ml; Peprotech, USA) and treated with or without butyrate (1 mM; Sigma Aldrich, Missouri, USA) in Roswell Park Memorial Institute media (RPMI; Gibco, Auckland, New Zealand) supplemented with fetal calf serum (FCS; Moregate, NZ), 2-mercaptoethanol (Sigma Aldrich) and Penicillin + Streptomycin (Gibco). In some experiments CD8+ T cells were treated with Mocetinostat (300 nM), TMP-195 (2.5 μM) or Trichostatin A (TSA; 10 nM; all from Selleck Chemicals, Texas, USA). For some experiments CD8+ T cells were pre-treated with Cell Trace Violet (CTV) proliferation dye (Thermofisher Scientific) before culture.

### *In vivo* tumor challenge

C57Bl/6J-Ptp mice received subcutaneous injection of 2 × 10^5^ B16-OVA cells in 50 μL phosphate buffered saline (PBS) into the right flank at day 0. At day 7 mice were adoptively transferred via intravenous injection with 5 × 10^4^ *in vitro* activated CD8+ OT-I T cells activated in the presence or absence of butyrate for three days in 200 μL PBS. Control mice received PBS alone. Mice were monitored regularly, and tumor area (measured as tumor length × tumor width) was tracked using calipers. Mice were euthanized 12 days after adoptive transfer of cells and spleen, tumor and tumor draining lymph nodes were extracted for T cell analysis.

### Cell isolation and flow cytometry

Mice were sacrificed via CO_2_ asphyxiation then spleens, tumors and tumor-draining lymph nodes were excised, and passed through sterile gauze and a 70 μm cell filter (Miltenyi Biotec, Macquarie Park, NSW, Australia). Tumor tissue was treated with Accutase (STEMCELL Technologies) at 37°C, 5% CO_2_ for 20 min before cell filtration. Single cell suspensions were then incubated for five h at 37°C, 5% CO_2_ with or without PMA (10 ng/mL), Ionomycin (500 ng/mL) and Brefeldin A (1 μg/mL; all from Sigma Aldrich). Cells were then treated with Zombie Fixable Live/Dead viability dye (BioLegend, San Diego, CA, USA) and 10 ug/mL TruStain FcX (anti-mouse CD16/32) antibody; (BioLegend) before being surface stained with fluorescent antibodies for 45 min at room temperature and fixed in 1% paraformaldehyde (Sigma Aldrich). Antibodies used were: anti-CD3 (clone 17A2), anti-CD8α (53–67), anti-CD25 (PC61), anti-IL-2 (JES6-5H4), anti-IFN-γ (XMG1.2), anti-TNF (MP6-XT22) anti-GzmB (NGZB), anti-T-bet (O4 46), anti-Eomes (X4-83), anti-CD44 (IM7), anti-CD62L (MEL-14), anti-KLRG-1 (2F1), anti-CD127 (S18006K), anti-CD122 [5H4 (for *in vitro*), TM-β1 (for *ex vivo*)], anti-PD-1 (J43), anti-CD5 (53–7.3), anti-CD28 (37.51), anti-Ki67 (SolA15), anti-CD45.2 (104), anti-TIM3 (RMT3-23), anti-Sca-1 (E13-161.7), H-K2B-SIINFEKL -Pentamer. All antibodies were purchased from Biolegend, BD Biosciences or Thermofisher. Pentamer was purchased from Proimmune. For Annexin V apoptosis staining, cells were treated with Annexin V AF647 as per manufacturer instructions (Thermofisher Scientific) before surface staining. For intracellular staining, fixed cells were treated with eBioscience permeabilization buffer (Thermofisher Scientific) before staining with intracellular fluorescent antibodies and analysis on a Cytek^®^ Aurora 4-Laser Ultraviolet-Violet-Blue-Red, Spectral Cytometer with SpectroFlo Software (Version 3.0, Cytek Biosciences, Freemont, CA, USA).

### Intracellular phospho-staining

Cells were rested in serum free RPMI media for 4 h then stained with Zombie NIR viability stain and 10 ug/mL TruStain FcX (anti-mouse CD16/32) antibody (BioLegend). Cells were then washed twice in ice-cold PBS, and treated with 50 IU of IL-2 for 1 min, 10 min or left untreated and incubated at 37°C. Reactions were stopped by adding pre-warmed (37°C) BD Cytofix buffer (BD Biosciences, Carlsbad, CA, USA) at a 1:1 ratio with cell suspension, then incubated for a further 10 min. Cells were then washed in ice cold PBS, before slow addition of 0.5 X BD Phosflow Perm Buffer IV (BD Biosciences) up to 1 mL and incubated at room temperature for 15 min. Fluorescent phospho-antibodies were added to cells and incubated at room temperature in the dark for 45 min. Cells were then washed twice in FACS buffer, before flow acquisition. Antibodies used were: anti-CD8 (clone 53–6.7), anti-p-STAT5a (pY694), anti-p-mTOR (MRRBY), anti-p-S6 (cupk43k), anti-p-AKT1 (SDRNR). All antibodies were purchased from Biolegend, BD Biosciences or Thermofisher.

### Flow cytometry and high dimensional cytometry analysis

Flow cytometry data was analyzed using FlowJo software (Version 10.8.1, FlowJo, LLC. Ashland, Oregon, USA). For high dimensional analyses, live, single-celled CD8+CD45.2+ T cell data was uploaded as FCS files onto OMIQ software (Dotmatics, Boston, Massachusetts).^1^ High dimensional analysis including Uniform Manifold Approximation and Projection (UMAP), CyCombine, Flow Self Organising Map (SOM) meta clustering and Significance Analysis of Microarrays (SAM) analysis and figures of these, were generated using OMIQ software.

### Statistical analysis

Statistical analysis is described in individual figure legends. Figures and statistics for most figures were generated using GraphPad Prism 10 (GraphPad Software Inc, San Diego, CA, USA). Statistical analysis was conducted without the assumption of normally distributed data, therefore non-parametric tests were used (Friedman, Wilcoxon, Kruskal Wallis) and *p*-values presented are derived from these tests. Where analyzed cells were from the same mouse, paired statistical analyses were performed. *P*-values of < 0.05 were considered significant. Results with *p*-values of up to 0.0625 were not considered statistically significantly different but were discussed in the text as indicative of a difference between groups.

## Results

### Butyrate induces an effector CD8+ T cell phenotype with enhanced effector function during *in vitro* T cell activation

**FIGURE 1:**
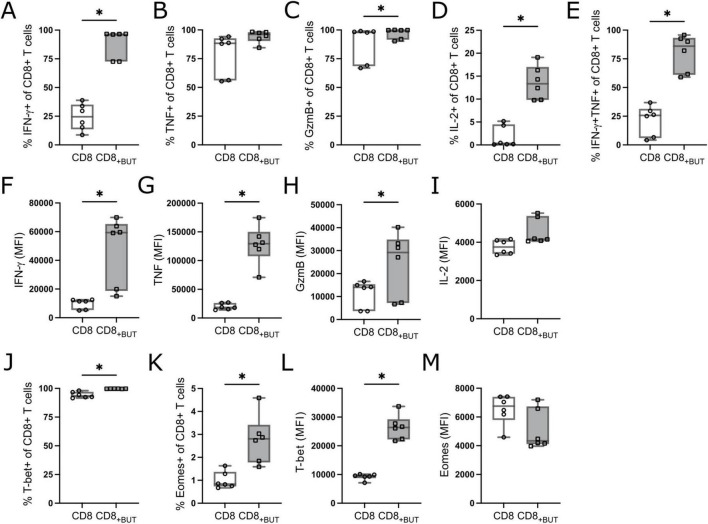
Butyrate enhances effector cell differentiation and function *in vitro*. OT-I CD8+ T cells were cultured with IL-2, anti-CD3 + anti-CD28 in the presence or absence of butyrate for three days then analyzed via flow cytometry. **(A)** Frequency of IFN-γ+ cells. **(B)** Frequency of TNF+ cells. **(C)** Frequency of GzmB+ cells. **(D)** Frequency of IL-2+ cells. **(E)** Frequency of IFN-γ+TNF+ cells. **(F–I)** MFI of IFN-γ, TNF, GzmB and IL-2. **(J)** Frequency of T-bet+ cells. **(K)** Frequency of Eomes+ cells. **(L,M)** MFI of T-bet and Eomes. Data show box plots of two independent experiments. *n* = 6 mice per group, Friedman test with Dunn’s multiple comparisons test. *p **≤** 0.05.

As previous work has shown that butyrate can enhance the frequency of effector CD8+ T cells *in vitro* (10, 11, 14), we first measured the frequency of CD8+ T effector-phenotype cells after butyrate treatment. OT-I CD8+ T cells were incubated with anti-CD3 + anti-CD28 activation beads and IL-2, in the presence or absence of 1 mM butyrate. After three days of culture, butyrate-treated cells had a statistically significantly greater frequency of IFN-γ+, GzmB+, IL-2+, and polyfunctional IFN-γ+TNF+ CD8+ T cells than untreated (no butyrate) cells (Figures 1A–E). The gating strategy is shown in Supplementary Figure 1. The median fluorescence intensity (MFI) of these markers was measured to infer the functional status of these T cells. Butyrate-treated cells had statistically significantly higher MFI of IFN-γ, TNF and GzmB than untreated cells (Figures 1F–H), potentially indicating superior function. Butyrate-treated cells had a statistically significantly higher frequency of T-bet+ and Eomes+ cells and MFI of T-bet, but not Eomes, than untreated cells (Figures 1J–M). After six days of culture, including removal of both activation beads and SCFAs, butyrate-treated cultures continued to display features of effector function and cell activation seen on day 3, including a high frequency of TNF+ cells, CD25+ cells and higher expression of GzmB and effector transcription factor T-bet than control cells (data not shown). Together, these results indicate that butyrate treatment enhances effector CD8+ T cell differentiation and improves effector function *in vitro*. These results may be mediated by enhanced activity of the transcription factor T-bet.

### Butyrate promotes CD8+ T effector and effector memory cell phenotypes during *in vitro* T cell activation

**FIGURE 2:**
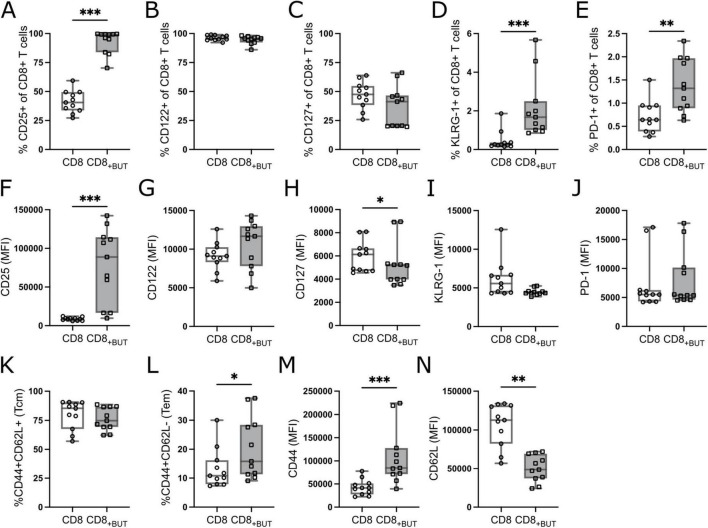
Butyrate increases activation of CD8+ T cells *in vitro*. OT-I CD8+ T cells were cultured with IL-2, anti-CD3 + anti-CD28 in the presence or absence of butyrate for three days then analyzed via flow cytometry. **(A)** Frequency of CD25+ cells. **(B)** Frequency of CD122+ cells. **(C)** Frequency of CD127+ cells. **(D)** Frequency of KLRG-1+ cells. **(E)** Frequency of PD-1+ cells. **(F–J)** MFI of CD25, CD122, CD127, KLRG-1, PD-1. **(K)** Frequency of CD44+CD62L+ (Tcm) cells. **(L)** Frequency of CD44+CD62L– (Tem) cells. **(M,N)** MFI of CD44 and CD62L. Data show box plots of five independent experiments. *n* = 11 mice per group, Friedman test with Dunn’s multiple comparisons test. *p ≤ 0.05, **p ≤ 0.01 ***p ≤ 0.001.

We next investigated the frequency of activation and memory-related markers to determine whether addition of butyrate during activation supported programming toward a memory phenotype. Butyrate-treatment resulted in a statistically significantly higher frequency of CD25+ cells than in untreated cells; however, no differences were observed in the frequency of memory markers CD122 (IL-2Rβ) or CD127 (IL-7Rα; Figures 2A–C). We also measured the frequency of the terminal differentiation marker Killer cell Lectin-like Receptor subfamily G1 (KLRG-1) and the T cell exhaustion/activation marker Programmed Cell Death Protein 1 (PD-1). Butyrate-treated cells had a statistically significantly higher frequency of both KLRG-1 and PD-1 than untreated cells (Figures 2D, E), which may indicate that butyrate promotes the generation of short-lived effector cells (SLECs). To determine if butyrate-treatment also altered the expression levels of these molecules, we measured the MFI of these markers. Butyrate-treated cells had statistically significantly higher MFI of CD25 and lower MFI of CD127 compared to untreated cells (Figures 2F, I). No differences were observed in the MFI of CD122, KLRG-1 or PD-1 between treatment groups (Figures 2H–J). Finally, we measured the frequency and MFI of the tissue homing receptor, CD44, and lymph homing receptor, CD62L, which together can be used to describe central and effector memory T cells (15). There were no differences in the frequency of central memory (CD44+CD62L+; Tcm) cells between treatment groups (Figure 2K). However, butyrate-treated cells had a statistically significantly higher proportion of effector memory (CD44+CD62L−; Tem) cells than untreated cells (Figure 2L). Butyrate-treated cells also had statistically significantly higher MFI of CD44 and lower MFI of CD62L compared to untreated cells (Figures 2M, N), both indicating highly activated cells. Combined, these findings suggest that butyrate improves the activation status of T cells and promotes both SLEC and T effector memory (Tem) phenotypes after three days.

### Butyrate reduces the rate of CD8+ T cell proliferation and increases apoptosis *in vitro*

**FIGURE 3:**
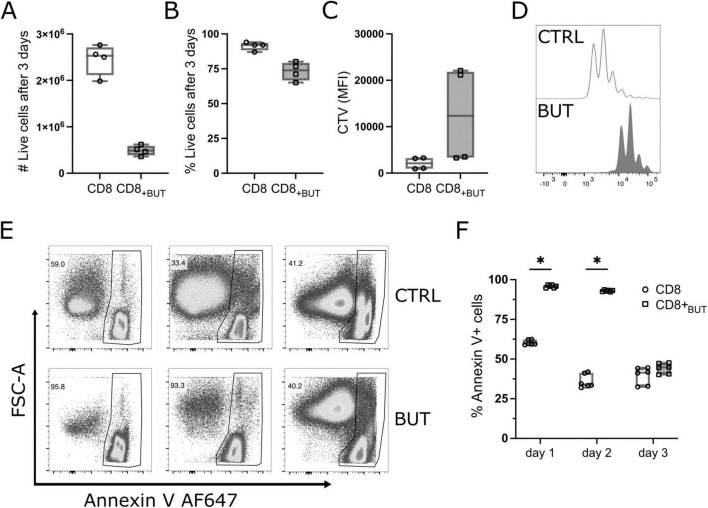
Butyrate reduces proliferation and promotes apoptosis of CD8+ T cells *in vitro*. OT-I CD8+ T cells were labeled with Cell Trace Violet (CTV) proliferation dye, cultured with IL-2, anti-CD3 + anti-CD28 in the presence or absence of butyrate for three days then analyzed via flow cytometry. **(A)** Total number of live CD8+ T cells. **(B)** Frequency of live CD8+ T cells. **(C)** MFI of CTV. **(D)** Histogram of proliferation peaks between control and butyrate -treated CD8+ T cells. **(E)** Representative flow cytometry plots showing FSC-A vs. Annexin V AF647 for control and butyrate-treated cells at day 1, 2 and 3 of culture. **(F)** Frequency of Annexin V+ cells at day 1, 2 and 3 of culture. Data show box plots of two independent experiments. *n* = 4–6 mice per group, Wilcoxon matched pairs signed rank test. *p < 0.05.

A hallmark of T cell activation is rapid clonal expansion. As butyrate-treated cells showed enhanced activation, we postulated that these cells would also have greater proliferation than untreated cells. We repeated the experiment described in Figure 1 while labeling T cells with Cell Trace Violet (CTV) to track proliferation. Unexpectedly, butyrate-treated cells had a lower total cell number and a lower frequency of viable cells after three days than untreated cells, although this difference was not statistically significant (Figures 3A–C). Butyrate-treated cells also underwent fewer rounds of proliferation than untreated cells (Figure 3D). We next explored if butyrate-treatment had toxic effects on cells, which could have reduced their cell number and viability. Annexin V levels were measured at 24, 48 and 72 h of culture to determine apoptosis. Butyrate-treated cells had a statistically significantly higher frequency of Annexin V+ cells at both 24 and 48 h than untreated cells (Figures 3E, F). These results show that butyrate-treatment may reduce the proliferation of CD8+ T cells and contribute to cell death at early timepoints after T cell activation.

Butyrate has a well-established role as a Histone Deacetylase (HDAC) inhibitor within T cells (10, 16–18). To determine if the HDAC activity of butyrate contributed to enhanced effector function and reduced cell viability, we compared the T cell response after butyrate-treatment to treatment with class I, II and pan HDAC inhibitors (Mocetinostat, TMP195 and TSA). CD8+ T cells were cultured with anti-CD3, anti-CD28, IL-2, and either butyrate or HDAC inhibitors for three days. T cells treated with Mocetinostat, but not TMP195 or TSA, had significantly higher frequencies and lower CD25 MFI of CD25+ cells than untreated cells but not butyrate-treated cells (Supplementary Figure 2). There were no significant effects of any HDAC inhibitor on the frequency of IFN-γ+ or TNF+ cells or MFI of these cells, nor of proliferation.

### Butyrate increases mTOR phosphorylation and expression of TCR molecules during *in vitro* T cell activation

**FIGURE 4:**
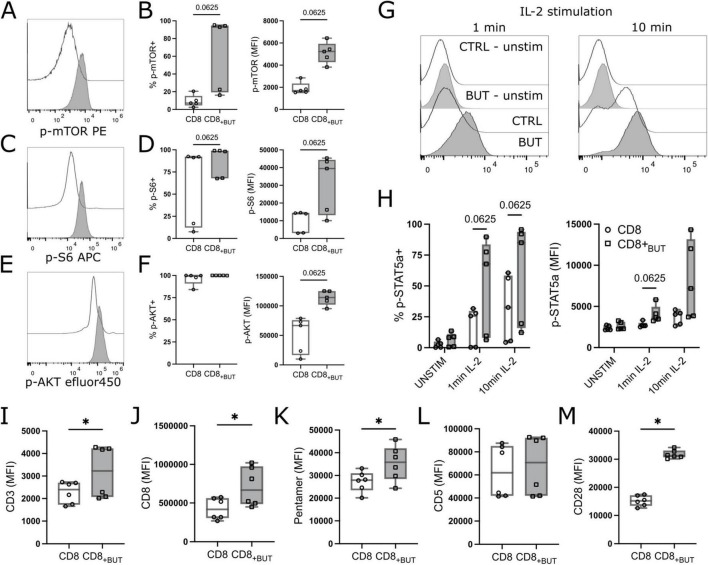
Butyrate enhances mTOR signaling, IL-2 signaling and expression of TCR molecules *in vitro*. OT-I CD8+ T cells were cultured with IL-2, anti-CD3 + anti-CD28 in the presence or absence of butyrate for three days. Cells were then serum starved and treated with IL-2 for 1 or 10 mins then analyzed via flow cytometry. [**(A,C,E)**; left] Representative histograms of p-mTOR, p-S6 or p-AKT MFI between butyrate-treated (blue) and control (gray) T cells. [**(A,C,E)**; right] frequency of p-mTOR+ p-S6+ or p-AKT+ T cells. **(B)** MFI of p-mTOR. **(D)** MFI of p-S6. **(F)** MFI of p-AKT. **(G)** Histograms of p-STAT5a between treatments after 1 min and 10 mins of IL-2 (50 IU) stimulation. **(H)** Frequency (left) and MFI (right) of p-STAT5a+ cells between treatment groups. **(I–M)** OT-I CD8+ T cells were cultured with IL-2, anti-CD3 + anti-CD28 in the presence or absence of butyrate for three days then analyzed via flow cytometry. MFI of CD3, CD8, H2^KB^ SIINFEKL pentamer, CD5 and CD28. Data show box plots of two independent experiments. *n* = 5–6 mice per group. Wilcoxon matched pairs signed rank test. *p < 0.05.

mTOR is part of a vital signaling pathway triggered after T cell activation (19). As butyrate-treated cells showed enhanced T cell activation, we asked whether butyrate-treated cells had enhanced mTOR signaling compared to untreated cells. Activated cells harvested after three days of culture in the presence or absence of butyrate were analyzed by phospho-flow cytometry for phosphorylated mTOR protein, ribosomal S6 protein (indicative of mTORC1 activation) and AKT1 at Ser473 (indicative of mTORC2 activation). Butyrate-treated cells showed a higher frequency of phospho(p)-mTOR+ and p-S6+ cells and higher MFI of all three proteins than untreated cells, although these differences were not statistically significant (Figures 4A–F).

As butyrate-treated cells also showed a significantly higher MFI of CD25 (Figure 2A), we then explored whether butyrate-treated cells exhibited enhanced IL-2 signaling by measuring downstream phosphorylation of STAT5a. Butyrate-treated cells had a higher frequency of p-STAT5a after both 1 min and 10 min of IL-2 stimulation, and MFI after 1 min, compared to untreated cells, although these differences were not statistically significant (Figures 4G, H). Finally, we also explored expression of TCR-related molecules. TCR activation is the initial step in T cell activation and promotes activation of mTOR signaling pathways. Butyrate-treated cells had statistically significantly higher MFI of CD3, CD8, H2^KB^ OVA (SIINFEKL) pentamer and CD28 compared to control cells (Figures 4I–K, M). There was no difference in CD5, a marker typically associated with the magnitude of TCR signaling expression between treatment groups (Figure 4L). Overall, these results indicate that butyrate-treated cells have enhanced mTOR signaling, IL-2 signaling and elevated expression of TCR-related molecules compared to untreated cells.

### *In vitro*-activated CD8+ T cells treated with butyrate enhance anti-tumor T cell responses *in vivo* following adoptive transfer

**FIGURE 5:**
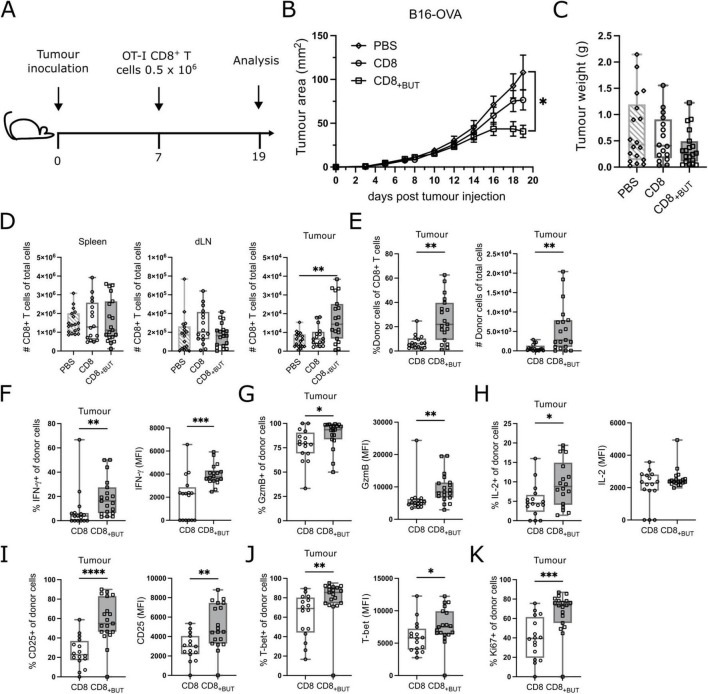
Butyrate-treated cells display enhanced *in vivo* tumor protection, effector function and proliferation. C57BL/6 Ptp mice received 2 × 10^5^ B16-OVA cells s.c at day 0 then 5 × 10^5^ OT-I CD8+ T cells *in vitro* activated ± butyrate i.v at day 7. At day 19 spleen, tumor and tumor draining lymph node were extracted and analyzed via flow cytometry. **(A)** Schematic for *in vivo* tumor experiments. **(B)** Tumor area (mm^2^) over time (days). **(C)** Tumor weights between treatments at day 19. **(D)** Total number of CD8+ T cells in the spleen, tumor draining lymph node and tumor. **(E)** Frequency and total number of donor CD45.2+CD8+ T cells within the tumor. **(F)** Frequency of IFN-γ+ donor cells and MFI of IFN-γ. **(G)** Frequency of GzmB+ donor cells and MFI of GzmB. **(H)** Frequency of IL-2+ donor cells and MFI of IL-2. **(I)** Frequency of CD25+ donor cells and MFI of CD25. **(J)** Frequency of T-bet+ donor cells and MFI of T-bet. **(K)** Frequency of Ki67+ donor cells. Data show box plots of three independent experiments. *n* = 15–19 mice per group, Kruskal–Wallis test with Dunn’s multiple comparisons test. *p ≤ 0.05 **p ≤ 0.01. ***p ≤ 0.001 ****p ≤ 0.0001. For **(B)**, statistical analyses were performed on the tumor area at day 19.

As butyrate-treated cells showed enhanced activation and effector function *in vitro*, we next explored if they conferred protection against tumor growth in mice. We used the subcutaneously administered B16 melanoma model of tumor immunity, which is widely used to study anti-tumor immune responses The B16-OVA cell line has been engineered to express the ovalbumin protein (OVA) in the context of MHC Class I, which allows analysis of SIINFEKL-specific CD8+ T cell responses as a proxy for an endogenous tumor associated antigen (20). C57Bl/6 (CD45.1+) mice were injected subcutaneously with B16-OVA melanoma cells, then seven days later received adoptive transfer of activated congenic OT-I CD8+ (CD45.2+) T cells that had been cultured for three days with or without butyrate *in vitro* as for previous figures. At day 19 after tumor inoculation, the tumor, spleen, and tumor draining lymph nodes were removed and analyzed using flow cytometry (Figure 5A; gating strategies shown in Supplementary Figures 3, 4). Mice that received butyrate-treated cells had statistically significantly lower tumor area compared to mice that received PBS only, but not untreated cells. There were no differences in the weight of tumors between groups (Figures 5B, C). There were no differences in the frequency of CD8+ T cells between groups in the spleen or draining lymph nodes; however, mice that received butyrate-treated cells had a statistically significantly greater frequency of CD8+ T cells within the tumor (Figure 5D) than mice that received either PBS or untreated cells. Furthermore, mice that received butyrate-treated cells also had a statistically significantly higher frequency and total number of CD45.2+ donor T cells in the tumors compared to mice that received untreated cells (Figure 5E). Together these results show that butyrate-treated cells can confer tumor protection in mice and this effect may be due to enhanced localization within the tumor.

To determine whether butyrate-treated cells displayed improved function *in vivo*, we investigated the effector function of donor cells recovered from the tumor. There was a statistically significantly higher frequency of IFN-γ+ and GzmB+ donor cells, and MFI of IFN-γ and GzmB in the tumors of mice that received butyrate-treated cells compared to those that received untreated cells (Figures 5F, G). There was also a statistically significantly higher frequency of IL-2+ donor cells in tumors of mice that received butyrate-treated cells compared to untreated cells; however, there was no difference in the MFI of IL-2 between these groups (Figure 5H). These results show that butyrate-treated cells display superior effector functions *in vivo*. Next, we measured the frequency of activation marker CD25 and transcription factor T-bet, and their MFI, which were both elevated after *in vitro* culture in the presence of butyrate (Figures 1, 2). There was a statistically significantly higher frequency of both CD25+ and T-bet+ donor cells, and a greater MFI of CD25 and T-bet, in butyrate-treated cells, compared to untreated cells, from the tumors of mice (Figures 5I, J). These findings indicate that butyrate-treated cells remain highly activated *in vivo* and express T-bet, a marker characteristic of effector T cells. Finally, we measured the frequency of Ki67+ cells to determine if donor cells were actively proliferating within the tumor. There was a statistically significantly higher frequency of Ki67+ donor cells in the tumors of mice that received butyrate-treated cells compared to mice that received untreated cells (Figure 5K). Together, these results demonstrate that butyrate treatment *in vitro* can improve the activation, effector function and proliferation of antigen-specific CD8+ T cells.

### *In vitro* butyrate-treated donor cells display features of effector memory T cells within the tumor environment *in vivo*

**FIGURE 6:**
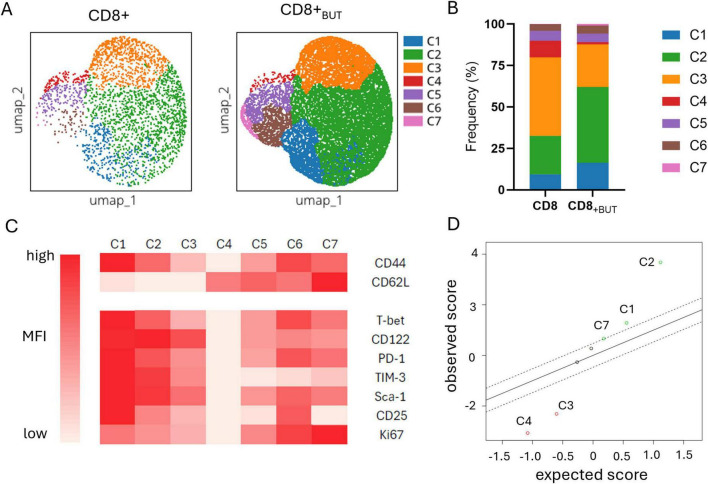
Butyrate-treated donor cells are enriched in activated effector T cells within the tumor. Data were obtained from the experiments shown in Figure 5. Fcs files of donor CD8+CD45.2+T cells previously cultured with or without butyrate and isolated from the tumor were uploaded onto OMIQ software. Files underwent data normalization, dimensionality reduction, elbow meta clustering before visualization on a clustered heatmap and SAM to determine differences in the abundance of clusters between butyrate-treated and untreated donor cells within the tumor. **(A)** UMAP plots showing the 7 meta clusters of donor cells within the tumor generated via FlowSOM. **(B)** Frequency of each meta cluster between donor cell treatments in the tumor. **(C)** Clustered Heatmap visualization of the MFI of markers within each donor cell cluster within the tumor. Cluster phenotypes: C1: CD44^hi^, CD62L^lo^, Tbet^hi^, CD122^hi^, PD-1^hi^, TIM-3^hi^, Sca-1^hi^, CD25^hi^, Ki67^med^; C2: CD44^hi^, CD62L^lo^, Tbet^med^, CD122^hi^, PD-1^med^, TIM3^hi^, Sca-1^hi^, CD25^med^, Ki67^med^; C3: CD44^med^, CD62L^lo^, Tbet^med^, CD122^hi^, PD-1^med^, TIM3^med^, Sca-1^med^, CD25^lo^, Ki67^lo^; C4: CD44^lo^, CD62L^hi^, Tbet^lo^, CD122^lo^, PD-1^lo^, TIM3^lo^, Sca-1^lo^, CD25^lo^, Ki67^lo^; C5: CD44^med^, CD62L^hi^, Tbet^med^, CD122^med^, PD-1^med^, TIM3^lo^, Sca-1^med^, CD25^lo^, Ki67^med^; C6: CD44^hi^, CD62L^hi^, Tbet^hi^, CD122^med^, PD-1^hi^, TIM3^lo^, Sca-1^hi^, CD25^hi^, Ki67^hi^; C7: CD44^med^; CD62L^hi^, Tbet^med^, CD122^med^, PD1^med^, TIM3^lo^, Sca-1^med^, CD25^lo^, Ki67^hi^. **(D)** Scatterplot of observed versus expected statistical difference in the abundance of each cluster between butyrate-treated and untreated donor cells within the tumor determined via SAM. Dotted lines show threshold cut offs for significance. Clusters above and below the dotted lines are significantly more (above) or less (below) abundant in butyrate-treated donor cells from the tumor compared to untreated donor cells from the tumor.

The previous figures characterized the effect of butyrate during activation using established knowledge of T cell populations and effector markers. Given the effect of butyrate in priming CD8+ T cells for anti-tumor function, we further characterized tumor-infiltrating donor T cells using high-dimensional analysis to determine the phenotype of populations enriched in the tumor, with a focus on effector and memory markers. We compared the phenotype of donor T cells from within the tumors of mice that received either butyrate-treated cells or untreated cells. Flow Self Organising Map (SOM) and elbow meta clustering was performed using the expression of only the memory markers CD44 and CD62L on donor cells. Seven cell clusters, based on differential expression of CD44 and CD62L, were identified and visualized using Uniform Manifold Approximation and Projection (UMAP; Figure 6A); the frequency of these clusters within butyrate-treated and untreated donor cells was compared (Figure 6B). The phenotype of each cluster was then determined by traditional cytometry for each of the activation, memory and exhaustion markers shown in the clustered heatmap (Figure 6C). The shading in the figure represents the relative expression of each marker within that cluster. To identify any clusters with statistically significant differences in their abundance between butyrate-treated and untreated donor cells, we performed Significance Analysis of Microarrays (SAM).

Figure 6D shows that Clusters 1, 2 and 7 were statistically significantly higher in abundance in the butyrate-treated cells than the untreated cells recovered from the tumors of mice. Clusters 1 and 2 displayed features of activated effector memory T cells (CD25+CD122+CD44+) including expression of both PD-1+TIM-3+ indicative of an active antigen-specific T cell response, while Cluster 7 displayed a central memory T cell phenotype with co-expression of CD44 and CD62L along with CD122 (Figure 6C). Clusters 3 and 4 were statistically significantly higher in abundance in untreated cells compared to butyrate-treated cells (Figure 6D). Cluster 3 also displayed an effector phenotype, but with lower median expression of all markers compared to Clusters 1 and 2, suggesting it may be a similar cell type as these clusters but at an earlier stage of activation. Cluster 4 displayed a naive T cell phenotype (CD44-CD62L+; Figure 6C). Clusters 5 and 6 displayed central memory features similar to Cluster 7. Cluster 6 showed high expression of CD25, PD-1, T-bet and Ki67 which may indicate they are activated, antigen-specific and actively proliferating. Cluster 5 showed similar marker expression but at lower levels also suggesting these clusters may represent the same cell type at different stages of activation. Despite this, there was no statistical difference in the abundance of either Cluster 5 or 6 between mice that received butyrate-treated cells versus untreated cells. Together, these findings show that butyrate-treated donor cell populations, with features of activated effector and central memory T cells, are enriched in tumor clusters compared to untreated donor cells.

Together, these findings show that butyrate-treated donor cell populations, with features of activated effector and central memory T cells, are enriched in tumor clusters compared to untreated donor cells.

## Discussion

This study shows that butyrate-treatment during murine T cell activation significantly enhanced not only the frequency of effector CD8+ T cells *in vitro* but also the amount of cytokines they produced and their overall activation. Importantly, butyrate-treated cells conferred anti-tumor protection *in vivo* and were enriched with effector and memory T cell populations in the tumor.

Butyrate increased the expression of the TCR, co-receptor CD8 and co-stimulatory protein CD28, which may improve the functional avidity of CD8+ T cells. Furthermore, butyrate enhanced mTOR signaling within CD8+ T cells as measured by phosphorylated mTOR, ribosomal S6 protein and Akt. These findings support previous work describing enhanced mTOR signaling after butyrate treatment (14, 16) and enhanced phosphorylation of intracellular signaling proteins downstream of the TCR after butyrate treatment (13). Results presented here suggest that increased signaling may occur via heightened upstream expression of TCR components. As mTOR signaling can also occur downstream of TCR engagement, enhanced TCR signaling may explain elevated p-mTOR levels in butyrate treated cells. Together, these mechanisms suggest that butyrate may act as an adjuvant within CD8+ T cells, “priming” them to recognize and respond to pathogens rapidly. Further support of this idea comes from elevated CD28 expression on butyrate-treated cells and augmented phospho-STAT5a protein within these cells after IL-2 stimulation, and from a recent study that showed butyrate interacted with toll-like receptor 5 (TLR5), a key pattern recognition receptor of flagellated bacteria.

There were high levels of apoptosis at 24–48 h after butyrate treatment, supporting previous findings (14, 17). Butyrate-induced Fas-dependent apoptosis through its activity as an HDAC 1 inhibitor has been previously described for CD4+ and CD8+ T cells (17). In our study, it is likely that differences in apoptosis also occurred via this mechanism, but this could be confirmed in future studies, as well as ruling out other potential mechanisms such as elevated pro-apoptotic proteins. A study by Li et al. (21) showed that pro-apoptotic Bcl-2 interacting mediator of cell death (BIM) protein expression was higher in CD8+ T cells with a high MFI of MHC pentamer. As our data showed a similar result, this finding could also be investigated. Furthermore, the expression of intracellular caspases could be explored at early time points. However, it may be more important to investigate the population of CD8+ T cells that resisted apoptosis after butyrate treatment. Indeed, resistance to apoptosis is typical of T cells with memory phenotypes. We did not assess the expression of activation markers of the apoptotic cells on Days 1–2 of culture, since these markers were not typically upregulated in the first two days of culture. However, it may be that some T cell populations in butyrate- versus untreated cells were more susceptible to apoptosis in the early stages of culture. It is possible that only CD8+ T cells with sufficient survival signals downstream of TCR signaling can overcome butyrate-induced apoptosis at early timepoints. Alternatively, CD8+ T cells with superior metabolic fitness, such as precursor memory cells, may survive by metabolizing butyrate, since butyrate can be directly used by CD8+ T cells as a fuel source (9). Consequently, this consumption may prevent intracellular accumulation of butyrate and promotion of Fas expression via HDAC inhibitor activity (17). In these cases, butyrate may act as a selection pressure for highly responsive, metabolically fit CD8+ T cells, such as memory CD8+ T cells. This result may explain why reduced butyrate levels have been seen in patients with colorectal cancer (12, 22). Without the selection pressure of butyrate, CD8+ T cells with reduced metabolic fitness and function may persist in the body leading to suboptimal anti-tumor responses. It is likely, however, that elevated mTOR levels in butyrate-treated cells improve their glycolytic activity, which is crucial for effector cell function (23). Studies by Bachem et al. (9) and Luu et al. (10) have both shown elevated glycolytic metabolism in butyrate-treated cells *in vitro*. Further, blocking glycolysis reduced production of IFN-γ in butyrate treated cells and treatment of CD8+ T cells with mTOR inhibitor rapamycin also reduced IFN-γ (10).

Mice transferred with butyrate-treated CD8+ T cells had a statistically significantly higher number and frequency of CD45.2+ donor cells within their tumor than mice transferred with untreated CD8+ T cells. This finding suggests that butyrate-treated CD8+ T cells may have superior tumor-homing capacity than untreated CD8+ T cells *in vivo*. Interestingly, there was not a statistically significant difference in tumor growth rate or final tumor weight between untreated and butyrate-treated cells. Given that there was a higher frequency of tumor-specific cells in the tumors of mice that received butyrate treated cells, this finding was surprising. It is possible that a difference in tumor growth may have become apparent at a later timepoint, since the growth curve for mice that received butyrate treated cells appeared to plateau after day 16. However, we were required to end the experiment according to our ethical approval protocol. Unlike Luu et al. (14), we did not see a significant difference in the number of butyrate treated cells in the draining lymph nodes. Butyrate-treated cells had higher median expression of CD44, a key molecule involved in migration of T cells into tissue than untreated cells (15). Additionally, high dimensional analysis revealed that mice transferred with butyrate-treated cells had statistically significantly higher abundance of donor cell clusters with high median expression of CD44 than mice transferred with untreated cells. This superior homing capacity of butyrate-treated cells may have enabled them to arrive within the tumor and elicit anti-tumor effects earlier than untreated cells, resulting in a reduced tumor area by day 19. This result may be supported by He et al. (11), who showed that 12 h activated OT-I T cells treated with butyrate had greater tumor infiltration and protection than untreated OT-I T cells when adoptively transferred into mice with EG7 lymphoma. He et al. (11) also found that butyrate-treated cells had enhanced expression of ID2, which, when knocked down, reduced the expression of chemokine receptor CX3CR1. Our work further suggests that butyrate may impact the homing activity of antigen-specific CD8+ T cells and should be investigated in future studies.

Unexpectedly, while butyrate-treated cells maintained high CD62L expression after 3 days of *in vitro* activation, when transferred *in vivo*, most butyrate-treated cells had low expression of CD62L. This may suggest that butyrate-treated cells undergo more antigen-specific activation *in vivo* than untreated cells, that promotes the shedding of CD62L (24). As the frequency of donor cells within the draining lymph nodes and spleen was relatively low compared to the tumor, the tumor may be the primary activation site of butyrate-treated cells. Indeed, Thompson et al. (25) have shown that the tumor site can be a major site of tumor-specific T cell activation. Furthermore, in our study Tem clusters within tumors displayed cell activation markers CD25, TIM-3, and PD-1 and proliferation markers Ki67 that all suggest recent TCR activation.

This study demonstrates that the SCFA, butyrate, has strong therapeutic potential with the ability to enhance the frequency and function of effector CD8+ T cells. This makes butyrate highly desirable for T cell-based therapies like CAR T cell therapy. As butyrate-treated cells showed elevated TCR expression, as well as long term *in vivo* persistence as memory cells, butyrate may also have applications in vaccine design, particularly if it can be targeted to sites of T cell activation, such as the secondary lymphoid organs.

## Data Availability

The raw data supporting the conclusions of this article will be made available by the authors, without undue reservation.

## Footnotes

1
https://www.omiq.ai/
