# Supplementary material for: The microbial metabolite butyrate enhances the effector and memory functions of murine CD8+ T cells and improves anti-tumor activity
Source: Front Med (Lausanne). 2025 Jun 24;12:1577906. doi: 10.3389/fmed.2025.1577906 (PMC12234554; doi:10.3389/fmed.2025.1577906)
Supplement: Supplementary file 1 [file Data_Sheet_1.pdf]

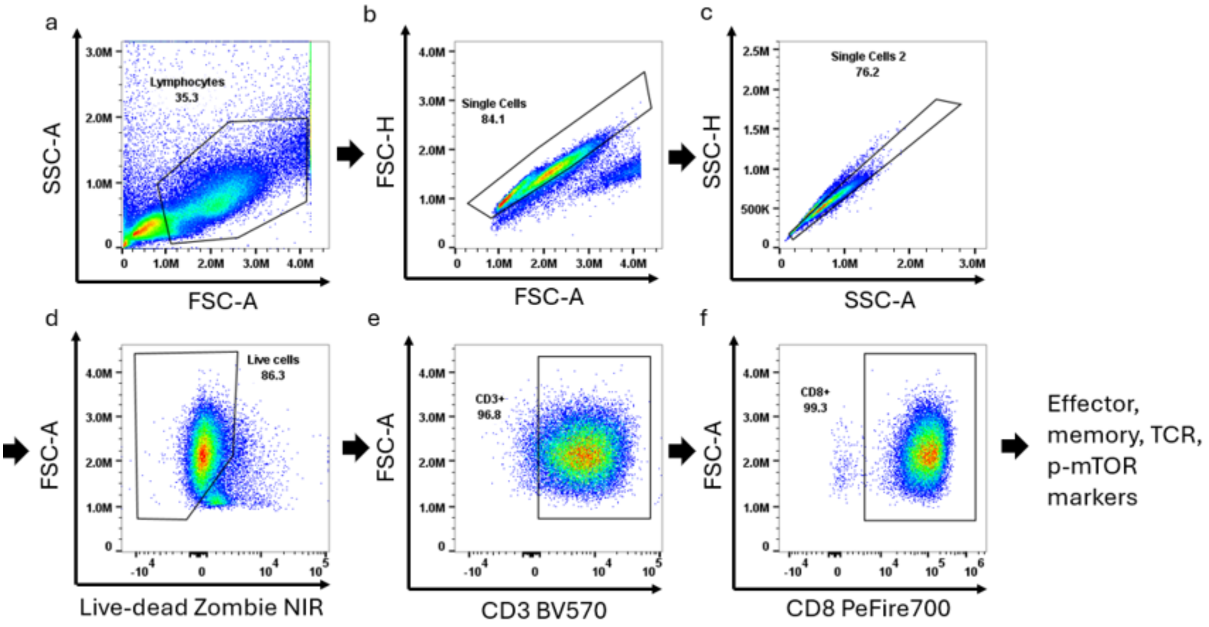

Supplementary Figure 1 : Representative gating strategy for CD8+ T cell analysis of effector, memory, and TCR markers. For p-Mtor experiments gating strategy did not include CD3 gate. (a) FSC-A vs SSC-A profile for gating of lymphocytes (b) Single cell gating based on FSC-A vs FSC-H (c) Single cell gating based on SSC-A vs SSC-H (d) Gating of live cells based on low staining for Zombie NIR viability dye (e) gating of CD3+ cells (f) gating of CD8+ T cells. All markers were gated directly downstream of CD8 gate. Example above is from cells treated in vitro with IL-2, anti-CD3 and anti-CD28, and butyrate for 3 days.
