# Supplementary material for: The microbial metabolite butyrate enhances the effector and memory functions of murine CD8+ T cells and improves anti-tumor activity
Source: Front Med (Lausanne). 2025 Jun 24;12:1577906. doi: 10.3389/fmed.2025.1577906 (PMC12234554; doi:10.3389/fmed.2025.1577906)
Supplement: Supplementary file 2 [file Data_Sheet_2.pdf]

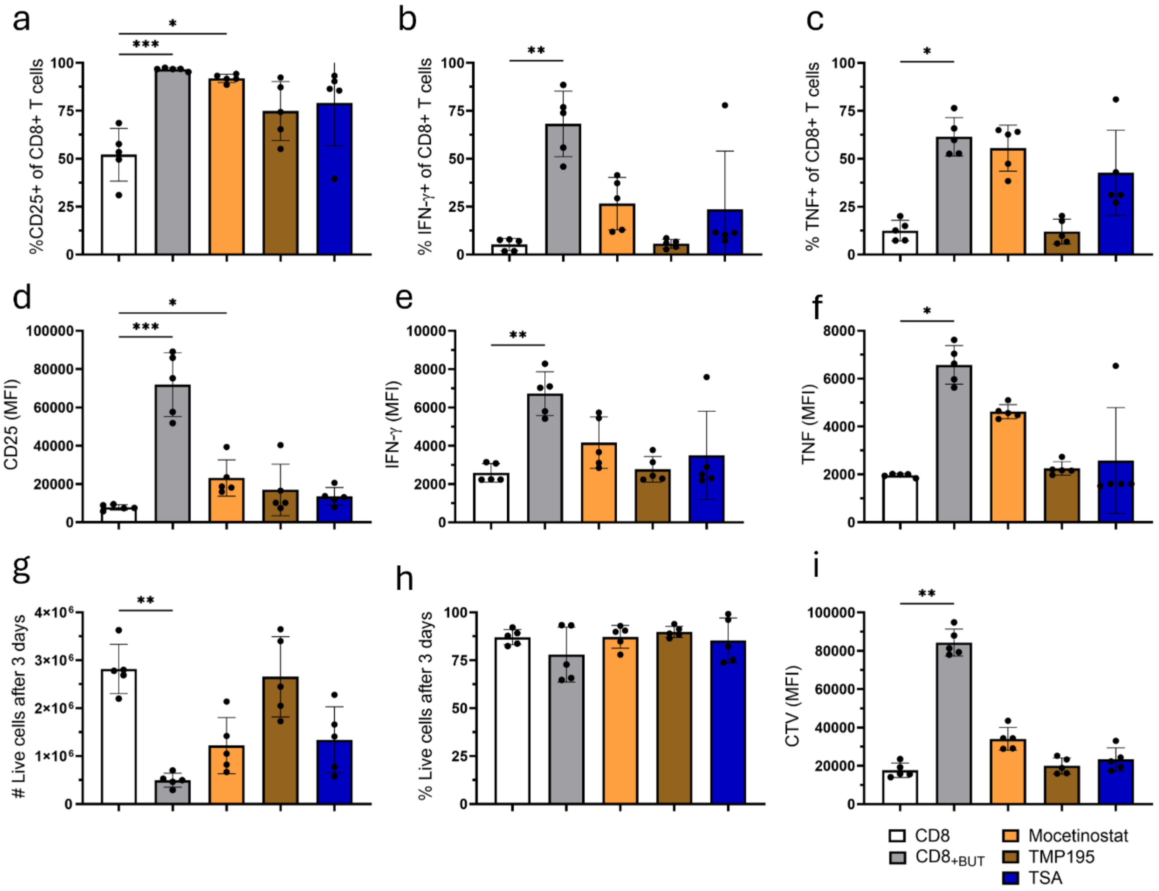

Supplementary Figure 2: HDAC inhibitor Mocetinostat increases the frequency and MFI of CD25. OT-I CD8+ T cells were labelled with Cell Trace Violet (CTV) proliferation dye, cultured with IL-2, anti-CD3 and anti-CD28 +/- either butyrate, Mocetinostat, TMP195 or TSA for 3 days then analysed via flow cytometry. (a-c) Frequency of CD25+ cell, IFN-γ+ cells and TNF+ cells between treatments.(d-f) MFI of CD25, IFN-γ and TNF. (g) Total number of live cells between treatments after three days of culture (h) Frequency of live cells between treatments after three days of culture. (i) MFI of CTV between treatments. Data show mean + SEM, the result of two independent experiments, n = 5 mice per group. Friedman test with Dunn's multiple comparisons test. *p = <0.05, **p = <0.01, ***p=<0.001
