# Supplementary material for: The microbial metabolite butyrate enhances the effector and memory functions of murine CD8+ T cells and improves anti-tumor activity
Source: Front Med (Lausanne). 2025 Jun 24;12:1577906. doi: 10.3389/fmed.2025.1577906 (PMC12234554; doi:10.3389/fmed.2025.1577906)
Supplement: Supplementary file 3 [file Data_Sheet_3.pdf]

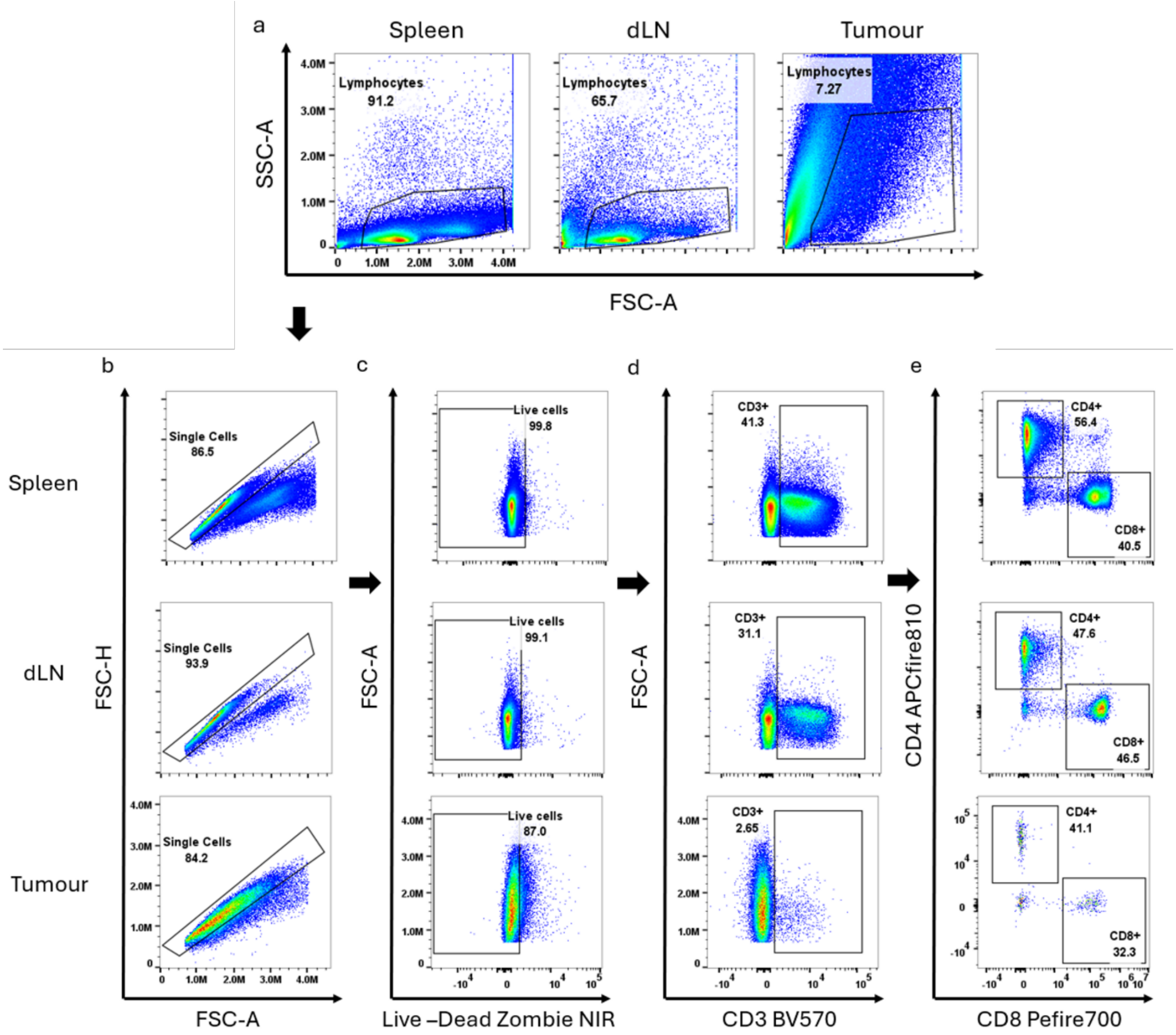

Supplementary Figure 3 : Representative gating strategy for ex vivo CD8+ T cell analysis. Spleen, tumour draining lymph node and tumour were isolated from Ptp mice 20 days after tumour injection. Tissues were processed into single cell suspensions then treated with fluorescent antibodies and analysed via flow cytometry (a) Gating of lymphocytes based on FSC-A vs SSC-A profiles. (b) Gating of single cells based on FSC-A vs FSC-H profiles. (c) Gating of live cells based on Zombie NIR viability dye vs FSC-A. (d) Gating of CD3+ T cells based on CD3 BV570 vs FSC-A. (e) Gating of CD8+ T cells based on CD4 APCfire810 vs CD8 Pefire700. Results show the spleen, tumour draining lymph node and tumour cells from one control mouse which received intravenous PBS at day 7 after tumour injection.
