# Supplementary material for: The microbial metabolite butyrate enhances the effector and memory functions of murine CD8+ T cells and improves anti-tumor activity
Source: Front Med (Lausanne). 2025 Jun 24;12:1577906. doi: 10.3389/fmed.2025.1577906 (PMC12234554; doi:10.3389/fmed.2025.1577906)
Supplement: Supplementary file 4 [file Data_Sheet_4.pdf]

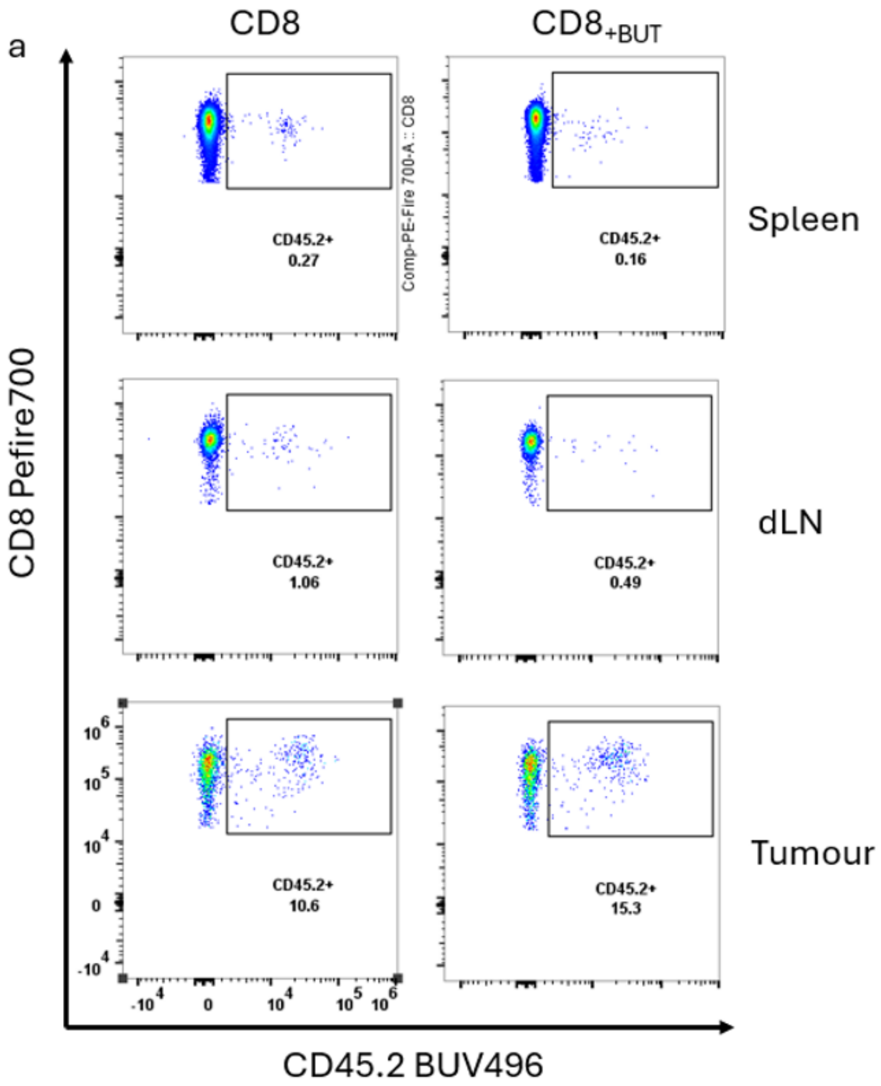

Supplementary Figure 4 : Representative gating for donor CD45.2+CD8+ T cells *ex vivo*. Spleen, tumour draining lymph node (dLN) and tumour were isolated from Ptp mice 20 days after tumour injection. Tissues were processed into single-cell suspensions, treated with fluorescent antibodies and analysed via flow cytometry. Cells were pre-gated to CD8+ T cells according to the gating strategy in supplementary figure 3 (a) Gating of CD45.2+ cells from the spleen (top), tumour draining lymph node (middle) and tumour (bottom) of mice transferred with activated OT-I CD8+ T cells treated with or without butyrate.
